# Weakly Correlated Local Cortical State Switches under Anesthesia Lead to Strongly Correlated Global States

**DOI:** 10.1523/JNEUROSCI.0123-22.2022

**Published:** 2022-11-30

**Authors:** Ethan B. Blackwood, Brenna P. Shortal, Alex Proekt

**Affiliations:** ^1^Department of Neuroscience, Perelman School of Medicine, University of Pennsylvania, Philadelphia, Pennsylvania 19103; ^2^Department of Anesthesia and Critical Care, Perelman School of Medicine, University of Pennsylvania, Philadelphia, Pennsylvania 19103

**Keywords:** anesthesia, oscillations, state transitions, synchrony

## Abstract

During recovery from anesthesia, brain activity switches abruptly between a small set of discrete states. Surprisingly, this switching also occurs under constant doses of anesthesia, even in the absence of stimuli. These metastable states and the transitions between them are thought to form a “scaffold” that ultimately guides the brain back to wakefulness. The processes that constrain cortical activity patterns to these states and govern how states are coordinated between different cortical regions are unknown. If state transitions were driven by subcortical modulation, different cortical sites should exhibit near-synchronous state transitions. Conversely, spatiotemporal heterogeneity would suggest that state transitions are coordinated through corticocortical interactions. To differentiate between these hypotheses, we quantified synchrony of brain states in male rats exposed to a fixed isoflurane concentration. States were defined from spectra of local field potentials recorded across layers of visual and motor cortices. A transition synchrony measure shows that most state transitions are highly localized. Furthermore, while most pairs of cortical sites exhibit statistically significant coupling of both states and state transition times, coupling strength is typically weak. States and state transitions in the thalamic input layer (L4) are particularly decoupled from those in supragranular and infragranular layers. This suggests that state transitions are not imposed on the cortex by broadly projecting modulatory systems. Although each pairwise interaction is typically weak, we show that the multitude of such weak interactions is sufficient to confine global activity to a small number of discrete states.

**SIGNIFICANCE STATEMENT** The brain consistently recovers to wakefulness after anesthesia, but this process is poorly understood. Previous work revealed that, during recovery from anesthesia, corticothalamic activity falls into one of several discrete patterns. The neuronal mechanisms constraining the cortex to just a few discrete states remain unknown. Global states could be coordinated by fluctuations in subcortical nuclei that project broadly to the cortex. Alternatively, these states may emerge from interactions within the cortex itself. Here, we provide evidence for the latter possibility by demonstrating that most pairs of cortical sites exhibit weak coupling. We thereby lay groundwork for future investigations of the specific cellular and network mechanisms of corticocortical activity state coupling.

## Introduction

Oscillations recorded from the brain reflect the evolution of a dynamical system composed of billions of interacting neurons and synapses, each of which itself exhibits complex nonlinear dynamics (Hodgkin and Huxley, 1952; Canavier et al., 1993; Pan and Zucker, 2009). Generically, complex nonlinear dynamical systems can dramatically and stably change their behavior after perturbations and small changes to their parameters (Destexhe et al., 1994; Ermentrout, 1998; Izhikevich, 2007; Strogatz, 2015). Therefore, it should be surprising that, even after dramatic perturbations, the brain consistently returns to its normal regimen of activity. For instance, after seizures, which are characterized by extreme synchronization in both neuronal firing and subthreshold voltage (Timofeev et al., 2004), normal brain function generally returns within hours (Fisher and Engel, 2010). Another dramatic perturbation of brain activity is anesthesia (Contreras and Steriade, 1997; Amzica, 2009; Civillico and Contreras, 2012). Millions of patients undergo general anesthesia each year and eventually recover normal brain activity and cognitive function. Since anesthetic delivery can be precisely controlled, general anesthesia is an ideal model system to investigate how the brain returns to normal activity after a dramatic perturbation.

During recovery from anesthesia, the firing patterns of neurons throughout the brain must eventually return to their preanesthetic state. The recovery process can be visualized as a path through a state space spanned by all possible activity patterns. While this space is potentially very high-dimensional, it has been shown that recovery paths are constrained to a low-dimensional subspace and involve abrupt transitions between a small number of stabilized activity patterns (Hudson et al., 2014). These constraints serve as a scaffold that helps the brain efficiently navigate through the high-dimensional space toward recovery. Discrete activity states and abrupt transitions between them have been observed in rodents (Hudson et al., 2014; Lee et al., 2020), nonhuman primates (Ishizawa et al., 2016; Ballesteros et al., 2020; Patel et al., 2020), and human patients (Chander et al., 2014) after exposure to a variety of anesthetics.

Given that abrupt changes in activity patterns are critical for reinstating consciousness, it is of fundamental importance to determine the neuronal mechanisms that underlie state transitions during recovery. Previous work on anesthesia (Chander et al., 2014; Hudson et al., 2014; Ishizawa et al., 2016) and sleep (Gervasoni et al., 2004) defined activity patterns based on oscillations observed in local field potentials (LFPs). It is well known that much of this oscillatory activity is coordinated via thalamocortical loops (Steriade et al., 1993b; Contreras and Steriade, 1997; Schiff, 2008; Liu et al., 2015). Furthermore, arousal pathways ascending from the brainstem and basal forebrain are known to modulate thalamocortical oscillations (Steriade et al., 1993a; Destexhe et al., 1994; B. E. Jones, 2003). If these ascending inputs from the thalamus, brainstem, and/or basal forebrain were solely responsible for driving cortical state transitions, we would expect that abrupt transitions between distinct oscillations would be synchronized across cortical layers and regions. Alternatively, it is possible that activity in different cortical regions is coordinated through short-range corticocortical interactions. In this case, transitions would be less synchronized.

Here, we provide direct experimental evidence for decentralized coordination of neuronal activity by identifying oscillatory states and transitions between them across cortical layers and distant cortical areas at a constant anesthetic concentration. We show that the synchrony of state transitions and coordination of states themselves are only weakly coupled between cortical sites. Furthermore, we demonstrate that states and transitions in the thalamic input layer (L4) are particularly decoupled from those observed in other layers. Finally, we show that the multitude of weak pairwise interactions between local state transitions constrains overall brain activity to just a few states embedded in a low-dimensional space. Thus, our results suggest that the highly coordinated, low-dimensional macroscopic brain dynamics that allow the brain to recover from a dramatic perturbation emerge from many weak pairwise interactions between different cortical sites.

## Materials and Methods

### Experimental design and statistical analyses

LFP recordings were obtained from 2 cohorts of male rats: 4 with linear probes inserted into right M1 and V1 and 6 with probes inserted into left and right V1. Recordings from 3 M1/V1 rats and 4 bilateral V1 rats were retained for all analyses (for exclusion criteria, see Animals). Each linear probe contained 64 recording sites spanning the entire thickness of the cortex (see Surgery). Within each animal, LFP from up to 10 sites on each probe were retained for analysis (see CSD and channel selection).

Three complementary measures of coupling, transition synchrony, normalized mutual information (NMI), and canonical correlation, were computed on all pairs of recording sites. Transition synchrony measures the propensity of two sites to switch their states at the same time. NMI measures the overall similarity between sequences of states at two sites. Canonical correlation measures the similarity between the spectra of two sites without assuming clustering of the spectra into distinct states. The same statistical analyses were applied to all three measures (see Statistical tests). These included individual, Bonferroni-corrected tests of whether the measure on each pair of sites was greater than predicted by a null model, as well as permutation- and bootstrap-based tests of differences of the means of specific groups of pairs.

### Animals

All experiments were performed using 10 male Sprague Dawley rats, each 2-3 months of age (250-350 g) (Charles River Laboratories). Two animals were excluded from further analyses because of excessive burst suppression or noise. One additional animal was excluded after current source density (CSD) analysis revealed that the V1 probe was inserted too deeply to clearly identify cortical L4 and the supragranular layers. Rats were housed under a conventional 12:12 h light:dark cycle and given food and water *ad libitum*. All experiments were performed in accordance with the Institutional Animal Care and Use Committee at the University of Pennsylvania and the National Institute of Health Guidelines.

### Surgery

All surgeries were performed under aseptic conditions. Animals were induced with 2.5% isoflurane in oxygen via a nose cone and secured in a stereotaxic frame (Kopf Instruments) in the prone position. Core body temperature was maintained at 37 ± 0.5°C using a temperature controller (TC-1000 Temperature Controller, CWE). Before surgery, isoflurane concentration was reduced to 1.5% (flow rate 1 L/min), and dexamethasone (0.25 mg/kg) was delivered subcutaneously to reduce the severity of cerebral edema (Holtmaat et al., 2009; Nimmerjahn, 2012). Bupivacaine (1 ml, 5 mg/ml) was injected under the scalp to provide local anesthesia (Hansen et al., 2013; University of British Columbia, 2015). Throughout the surgery, the lack of response to a toe pinch was used to assess proper anesthetic depth.

The scalp was retracted, and two 2 × 2 mm craniotomies were performed using a dental drill: one centered over −5.52 mm AP, 4 mm ML of bregma and another centered over −1.26 mm AP and 1.55 mm ML of bregma for V1 and M1 sites, respectively. Dura was removed, and Gelfoam (Pfizer) was placed on the exposed cortical tissue to prevent the tissue from desiccating. A reference screw was inserted 2 mm anterior and 1 mm to the left of bregma. Before insertion, both linear probes (Cambridge NeuroTech; H3 acute 64-channel linear probe) were dipped in DiI to permit subsequent track tracing and lowered to 1.2 mm into the brain at 2 µm/s. Before electrode insertion, Dura Gel (Cambridge NeuroTech) was applied to each craniotomy, and the isoflurane concentration was lowered to 1% (flow rate 1 L/min) for recordings. After the signals were deemed satisfactory, brief LED light stimuli were delivered to aid layer identification through offline CSD analysis (see CSD and channel selection). Between 30 and 60 min elapsed between electrode implantation and the beginning of the recording session to allow brain parenchyma to relax around the electrode. Immediately following electrophysiological recordings, animals were deeply anesthetized with 4% isoflurane and perfused transcardially with 4% PFA. Fixed brains were harvested and processed for electrode localization.

### Histologic confirmation of recording sites

Brains were sectioned at 80 µm on a vibratome (Leica Microsystems). Sections were mounted with medium containing a DAPI counterstain (Vector Laboratories). Electrode tracks were manually identified and localized using epifluorescence microscopy (Olympus; BX41) at 4× magnification.

### Electrophysiology and preprocessing

All recordings were performed in rats breathing 1% isoflurane in oxygen. Signals were amplified and digitized on an RHD2132 headstage (Intan) and streamed to a PC using an Omniplex acquisition system (Plexon) at a rate of 40,000 samples per second per channel. All recordings were performed using a ground skull screw as reference. LFPs were extracted from raw signals online using the bandpass filter with a passband of 0.1-300 Hz. Offline, LFPs were decimated to 1 kHz and filtered using a custom acausal FIR 0.1-200 Hz bandpass filter. Noisy channels were removed by visual inspection of the signals. Before subsequent analyses, LFPs were rereferenced to the mean computed over all clean channels on the laminar probe. An example 5 min segment of rereferenced LFP is shown in Figure 3*A*. All data analysis was completed using custom-built MATLAB (The MathWorks) code unless otherwise stated. In total, 29.88 h of recordings were used to generate all data in this manuscript. Recording durations from single rats included in analyses ranged from 4 to 7.25 h, with a median of 5.5 h per animal.

### CSD and channel selection

The characteristic response to a visual stimulus in the thalamic input layer of V1 was used as the basis to identify all cortical layers. At the start of the first recording on each day, green LEDs positioned 1 inch from each eye contralateral to a V1 recording each fired a series of 10 ms light flash stimuli. Interstimulus intervals were sampled from the uniform distribution between 3 and 5 s to prevent stimulus entrainment. The CSD *C_t_* was then computed from the mean post-stimulus LFP *V_t_*, using a smoothed second spatial derivative kernel *K* (a representative example is shown in Fig. 6) as follows:

$$\begin{array}{l} C_{t}(z)=V_{t}(z)*K(z)\\ K(z)=\frac{z^{2}-\sigma^{2}}{\sigma^{5}\sqrt{2\pi }}\exp\left(\frac{-z^{2}}{2\sigma^{2}}\right) \end{array}$$

Here, *z* is the channel depth, σ = 280 μm is the distance along the electrode from *z* at which the kernel changes sign, and * indicates convolution. The electrode closest to the center of L4 was identified manually from the CSD as the earliest current sink. Once L4 was identified, supragranular and infragranular channels were selected for analysis at 140 μm intervals above and below L4 because volume conduction is on the order of hundreds of micrometers (Maier et al., 2010; Kajikawa and Schroeder, 2011).

### Time-frequency analysis

Spectrograms of selected channels were obtained from LFPs using the Thomson multitaper method, which yields robust spectral estimates given finite data segments (Percival and Walden, 1993). A 6 s sliding window with a step size of 100 ms was used (17 Slepian tapers and time-bandwidth product (*NW*) = 9). Windows containing signal artifacts were identified and removed using a combination of a custom automatic burst suppression detection algorithm and manual inspection. To sample frequencies of interest more densely, each window was zero-padded to 2^16^ samples, and then *F* = 279 frequencies were selected from the output, spaced on a log scale from 0.14 to 10 Hz and on a linear scale from 10 to 300 Hz. The spectrograms were then smoothed over frequencies with a median filter spanning 10 frequency steps (up to 17.5 Hz) and over time with an exponential (Poisson) window spanning 2 min. To remove baseline differences in power across frequencies (e.g., power-law scaling) and emphasize temporal fluctuations, each spectrogram was rank-order normalized along the time axis. At each frequency bin, the time window with the highest power was given the value of 1. Each other window was given the value of (*r* − 1)*/*(*N* − 1), where *r* is that window's sorted index among the *N* windows. Thus, the smallest power value at each frequency was represented as 0 and the largest as 1. The full process had a time resolution of *t_R_* ≈ 5.9 s, in the sense that pairs of impulses spaced *t_R_* or greater apart would produce separate peaks in the normalized spectrogram. An example 5 min segment of a normalized spectrogram is shown in Figure 3*B*.

### Dimensionality reduction

The spectrograms were reduced from *F* = 279 frequencies to *K* ≈ 10 components to further remove redundancy and focus on differences across time. Dimensionality reduction was performed on each channel's spectrogram individually, to ensure that any characteristic differences in activity patterns between sampled regions and cortical depths were preserved. Non-negative matrix factorization (NMF) (Lee and Seung, 1999; Mankad and Michailidis, 2013) was used, which represents the signal at each time as a vector of *K* non-negative coefficients (scores) that weight a sum of corresponding frequency components (loadings) to reproduce the original spectrum. Given a spectrogram *A* of size *F* × *N*, NMF produces a loading matrix *U* of size *F* × *K* and a score matrix *V* of size *N* × *K*, such that *UV*^T^ ≈ *A*. The reconstruction error *E* is quantified relative to the norm of *A* as follows:

$$E=\frac{{\left\|A-UV^{T}\right\|}_{F}}{{\left\|A\right\|}_{F}}$$

Where ${\left\|\cdot \right\|}_{F}$ is the Frobenius norm. To select an appropriate number of components (*K*) for each channel, a cross-validation approach was used (Owen and Perry, 2009). First, spectrograms were downsampled across time by a factor of 20, for computational efficiency. Then, a random subset of 20% of the rows and columns were selected to be withheld. Starting with *K* = 1 and increasing to 15, NMF was applied to the downsampled matrix after the random subset of rows and columns had been removed. This iteration provides both a loading and score matrix. Next, NMF was run again on the data with only the preselected rows withheld. In this iteration, the loading matrix from the first round was fixed and only a new score matrix was calculated. In the third and final run of NMF, NMF was run on the data with only the preselected columns removed, fixing the score matrix from the first round and calculating only a new loading matrix. Finally, the loading and score matrices produced in the second and third run of NMF, respectively, were multiplied to generate an estimate of the original dataset and calculate error as a function of *K*. This procedure was repeated for five replicates for each value of *K*, and the optimal *K* was chosen such that increasing *K* by 1 would reduce mean reconstruction error by <1%. In our dataset, the optimal value for *K* ranged from 5 to 9 for different channels. After the cross-validation procedure, each channel's full, normalized spectrogram was subjected to NMF using the channel's optimal *K*, resulting in a mean reconstruction error of 14.8% across all channels (∼85% of the variance captured by NMF for each spectrogram). NMF does not constrain the relative scales of the loading vectors: for any invertible diagonal *K* × *K* matrix *D*, *UV*^T^ = *UD*(*VD*^−1^)^T^. To remove these degrees of freedom, *U* and *V* were rescaled by a matrix *D* such that the rescaled loadings had unit L_2_ norm. Figure 3 shows the results of NMF on an example recording, including the loading matrix *UD* (C), a 5 min segment of the score matrix *VD*^−1^ (D), and the normalized spectrogram reconstruction produced by their product (E).

### Transition and discrete state identification

The rescaled score matrix *VD*^−1^ is the basis for defining each channel's state over time. Figure 4*A* shows 5 min segments of these matrices for 2 of the 18 channels in an example recording. For each channel, at each time point, the component with the highest score was interpreted as the state, and samples where the state changed were marked as local transition times. To prevent an arbitrarily high number of transitions during periods when two or more components had similar scores, transitions that were likely to reflect transient fluctuations were ignored and the state assignments between them were updated accordingly. Specifically, suppose one time segment between two transitions was assigned state *A* and either the previous or next segment was assigned state *B*. If the first segment was <100 s long and, within the first segment, the mean score for NMF Component *A* was <1.1 times the mean score for Component *B* (i.e., if the state assignment was sufficiently ambiguous), the transition between the two segments was ignored and the combined segment was assigned State *B*. If a segment could be merged with either the previous or next segment, the tie was broken by ignoring the transition with a smaller magnitude of change in the full NMF score vector from the 3 s before the transition to the 3 s following it. Figure 4*B* shows the state assignments of all channels in the example 5 s segment. State transition frequencies were also computed by tabulating how often each discrete state followed each other state over the duration of the recording.

### Discrete state visualization and consistency analysis

To visualize the pattern of LFP most strongly associated with each discrete state in example channels, multitaper windows assigned to each state were ranked by the ratio of their top NMF score (corresponding to the assigned state) to the sum of all NMF scores. Each panel of Figure 5*A* shows the LFP of the highest- or second highest-ranked window according to this measure (chosen manually) within the indicated state. To visualize spectral changes accompanying state transitions, spectrograms before and after all transitions of the most common type in the analyzed channel were averaged. To assess the consistency of LFP patterns and state classification across animals, a method similar to Hudson et al. (2014, their Fig. S10) was used. In 500 independent repetitions, 600 multitaper spectra were drawn without replacement from each discrete state of channels at the same depth and in the same region across all animals, then concatenated into a combined dataset. This dataset was then classified into “mixed states” using the same methods listed above for individual-channel states. The NMI of these mixed state assignments with both the animal IDs and each constituent channel's state assignments was then computed (see NMI). The distribution of NMI with animal IDs was compared with that of the mean NMI with individual-channel states across animals. If the range of spectra being classified is similar across animals, and these spectra are split into states using a consistent scheme across animals, then mixed states should map to individual-channel states more closely than to animal IDs.

### Markov-shuffled null model

When testing whether pairs of channels are synchronized in the sense that certain pairs of states tend to occur together, apparent synchrony could arise because of the channels' individual NMF score distributions, independent of transition timing. To control for this possibility, a discrete-time Markov chain (the “null model”) was fit to the transition frequencies of each channel independently and used to simulate 1000 new discrete state sequences of the same length as the original data. For each pair of channels, these “null” state sequences were then used to fit distributions of transition synchrony and NMI (see corresponding sections below). This distribution reflects the probability of observing the actual coupling scores under the assumption of complete independence between different recording sites. To obtain a null distribution of canonical correlation-based synchrony (see below), full score matrices were generated from each channel's null state sequences as follows: for each of the *K* states *k*, at each sample with null discrete state assignment *k*, the corresponding row of the null score matrix was randomly drawn from the set of rows of the original data score matrix *V* at samples where the original discrete state was equal to *k.* These random sequences for all pairs of channels were then subjected to canonical correlation analysis (CCA).

After fitting normal distributions for each of the three channel pair coupling measures (transition synchrony, NMI, and canonical correlation) to the shuffled surrogates, the values obtained for the real data were tested against these distributions to estimate whether they would be expected by chance, given the statistics of the data (see Statistical tests).

### Transition synchrony

To evaluate how frequently the discrete states of pairs of channels changed together, we used the SPIKE-synchronization score (“synchrony score”) (Kreuz et al., 2015). At its core, this method is a coincidence detector in which the coincidence window is derived from the dataset. The adaptive definition of the coincidence window means that this method for quantifying synchrony is equally well suited for state transitions as it is to spike trains. Each transition *r* is assigned a local window length τ(*r*), which is defined as half the smaller of the intertransition intervals directly before and after *r*. For a pair of channels *i* and *j*, if transition *r_j_* in *j* was the closest transition to transition *r_i_* in *i* and vice versa, and the time between *r_i_* and *r_j_* is less than min(τ(*r_i_*), τ(*r_j_*)), both transitions have a synchrony score of 1. All other transitions have a score of 0. This measure is extended to the multichannel case by letting each transition's global synchrony score equal the mean of its pairwise synchrony scores with all other channels. Both pairwise and global synchrony scores were computed for all discrete state transitions in each recording, and then averaged over all transitions to obtain pairwise and global mean synchrony measures. Figure 4*C* shows a raster plot of all state transitions in the example 5 s segment, colored by their global synchrony scores.

### NMI

Mutual information of discrete states was used to quantify the synchrony of states themselves rather than just the timing of their transitions. Specifically, this measure was implemented to quantify how well one could predict the state in one channel, given the state of another channel at the same time point. Since NMF was performed separately on each channel, states labeled with the same index in different channels are not necessarily the same with respect to the frequency characteristics of the signal. Regardless, mutual information reveals temporal relationships between channel pairs because it does not assume any particular relationship between the state assignments of the different channels and is, therefore, agnostic to the assignments themselves.

Mutual information *I*(*X; Y*) between two channels *X* and *Y* with the same number of samples *N* and different sets of classes *K_X_* and *K_Y_* was computed pointwise as follows:

$$I(X;Y)=I(Y;X)=H(Y)-H(Y\mathrm{|X})H(Y)=-\sum_{k\in K_{Y}}P(Y=k)\log_{2}P(Y=k)H(Y\mathrm{|X})=-\sum_{j\in K_{X}}P(X=j)\sum_{k\in K_{Y}}P(Y=k|X=j)\log_{2}P(Y=k|X=j)P(Y=k)=\frac{\left|\left\{t|Y\left[t\right]=k\right\}\right|}{N}P(Y=k|X=j)=\frac{\left|\left\{t|X\left[t\right]=j,Y\left[t\right]=k\right\}\right|}{\left|\left\{t|X\left[t\right]=j\right\}\right|}$$

Mutual information is not a pure measure of the predictability of one variable given the other; it also increases with the entropy of each variable. For example, if channels *X* and *Y* each occupy a wider distribution of states and, as a result, have higher entropy than both channels *W* and *Z*, then *I*(*X*; *Y*) > *I*(*W*; *Z*). This is true even if the state of *X* is perfectly predictable given *Y*, *Y* given *X*, *W* given *Z*, and *Z* given *W*. In order to control for this, mutual information was normalized by the sum of the entropies of the two channels, giving the NMI, or symmetric uncertainty (Witten et al., 2011):

$$U(X,Y)=2\frac{I(X;Y)}{H(X)+H(Y)}$$

Using another definition for mutual information in terms of the individual and joint entropies of *X* and *Y*, we can write the following:

$$U(X,Y)=2\frac{H(X)+H(Y)-H(X,Y)}{H(X)+H(Y)}$$

Thus, NMI can be understood as twice the fraction of the sum of individual entropies, *H*(*X*) + *H*(*Y*), that exceeds (is redundant to) the joint entropy *H*(*X, Y*) because of mutual information between *X* and *Y*. For example, if *X* and *Y* are identical, *U*(*X, Y*) = 1 and 50% of *H*(*X*) + *H*(*Y*) is redundant, as only one of the variables carries unique information.

### Canonical correlation

Both the transition synchrony and NMI measures assume that LFP signals at each channel form discrete states and that the sequence of NMF components with the largest magnitude at each time point is informative about this state. However, there may be cases where multiple components must be considered. For instance, consider a situation in which NMF Component *A* in channel *i* is characterized by strong activity in two frequency bands, and Components *B* and *C* in channel *j* are characterized by strong activity in one of those frequency bands each. If only the “top” component determines the discrete state, there could be artificially low synchrony and mutual information between channels *i* and *j*. This is because, during a bout of State *A* in channel *i*, there could be frequent switching between States *B* and *C* in channel *j*, although the overall signal characteristics in channel *j* remain largely static. To address this kind of ambiguity and compute a state synchrony measure that softens the artificially sharp boundaries between “discrete states,” CCA was applied to the NMF score matrices of pairs of channels. Intuitively, CCA allows each score matrix to be linearly transformed to optimally match components between channels. CCA maximizes the correlations between the matched, transformed components. These correlations are used to derive a measure of state similarity.

The procedure for computing CCA-based synchrony is as follows: let *V* ∈ ℝ^*NxL*^ and *W* ∈ ℝ^*NxM*^ be the NMF score matrices two channels, and let *K* = min(*L, M*). At each step *i* from 1 to *K*, CCA finds coefficient vectors *a_i_* and *b_i_* to maximize the correlation ρ*_i_* = corr(*Va_i_*, *Wb_i_*), with the constraints that *a_i_* is uncorrelated with all previous vectors *a*_1_, …, *a_i_*_-1_, and likewise for *b_i_.* The MATLAB function *canoncorr* was used to perform this algorithm and the canonical correlation coefficients ρ_1_, …, ρ*_Κ_* were averaged to obtain a state similarity measure.

### Statistical tests

This section describes the procedure used to establish the statistical significance of interactions between recording sites, as measured by the synchrony score, NMI, and canonical correlation analyses. For each channel pair under consideration and each of these three coupling measures, the measure was computed both on the experimental dataset and on a set of 1000 null-model datasets generated from discrete Markov models of each channel's transition statistics, as described above. The values of each measure were approximately normally distributed across null-model datasets. To test statistical significance, the deviation of each measure obtained in the experimental dataset from those generated from null-model datasets was expressed as a *z* score. The one-tailed *p* value was then directly computed from the *z* score. The significance threshold was set at α = 0.05. Bonferroni correction was applied to account for multiple comparisons over all channel pairs in each animal. The percentage of pairs for which each coupling measure was different from chance after Bonferroni correction is reported in the manuscript, and nonsignificant pairs are grayed out in Figures 7–9.

To compare coupling measures between different sets of channel pairs, special consideration must be paid to the statistical dependence between observations. In a recording with *n* channels, for any channel *k*, one would not in general expect the values of a distance-like measure on the pairs (*k*, 1), …, (*k*, *k*−*1*), (*k*, *k* + 1), …, (*k*, *n*) to be independent. For example, if channel *k* were an outlier, all *n*-1 pairs would take extreme values because of what is statistically only one extreme observation. If pairwise statistics were compared naively (e.*g*., using a two-sample *t* test), these dependencies would result in an overestimation of effective sample size and thus significance. Instead, a Monte Carlo permutation procedure was used to establish null distributions for comparisons of pairwise measures between groups of channel pairs. This procedure randomly shuffled group assignments while preserving the dependency structure inherent in the matrix of pairwise measures by only shuffling rows and columns. For each such comparison, 10^7^ permutations of only the channels of each recording that were included in that comparison were conducted, and the difference of group means was computed after each permutation. The frequency with which these null differences exceeded the difference of means of the unpermuted groups was taken as the *p* value of the comparison.

Finally, when comparing the coupling measures for between-region channel pairs in M1/V1 recordings to those in bilateral V1 recordings, the method of permuting channel labels cannot be used because there are no data for pairs of channels that mix different recordings. Instead, the distribution for the difference of means of the measure over pairs between the two sets of recordings was estimated by bootstrapping over channels. Specifically, each group in such a comparison consists of a set of rectangular matrices, containing values of the measure for each pair of one channel along the rows and one channel along the columns. By resampling both rows and columns with replacement in each such matrix, the dependencies along rows and columns were preserved, but the variance in the mean could be estimated thanks to the principles of bootstrapping. A total of 10^6^ bootstrapped estimates of the group mean difference were computed in this manner for each coupling measure and used to obtain a *p* value for the one-tailed hypothesis that the measure is greater on average between hemispheres of V1 than between M1 and V1.

### Code accessibility

Code for all analyses is publicly available at https://github.com/ProektLab/spec-state-trans (for other public dependencies, see the README).

## Results

### State transitions under constant anesthetic can be local

**Figure 1.**
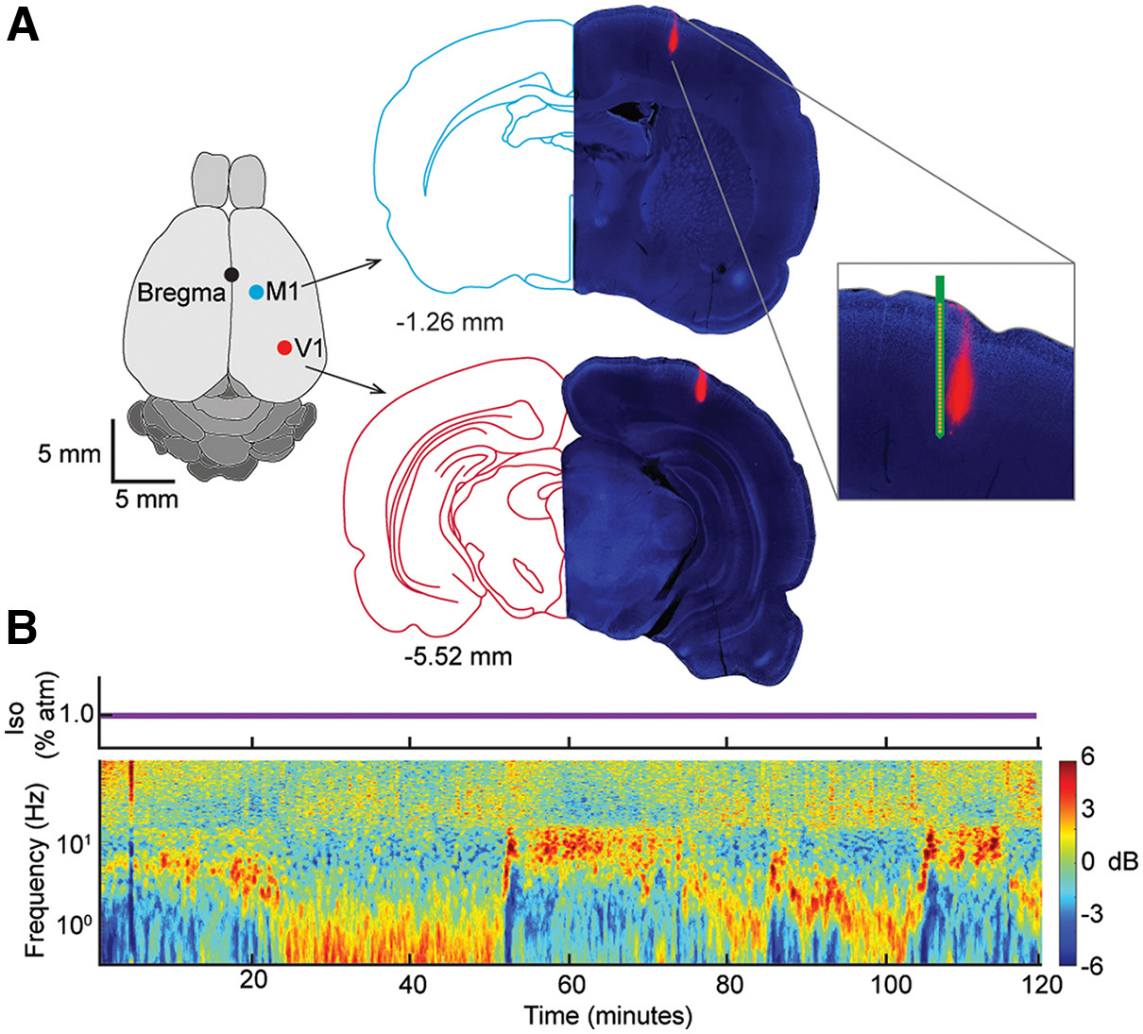
Experimental setup. ***A***, Verification of electrode placement into V1 and M1. DAPI-stained histologic section showing tracks of the DiI-dipped electrode (right) juxtaposed with the corresponding section from the rat brain atlas (left). The zoomed cutout includes an image to show electrode channel layout. ***B***, Time-resolved spectrogram recorded from V1 under 1% isoflurane general anesthesia (concentration shown above spectrogram). Spectrogram is plotted as deviations from temporal mean.

We sought to determine whether state transitions under a fixed concentration of isoflurane (1% atm) occur simultaneously across different cortical regions and across layers within the same cortical region. This concentration was chosen based on previous work (Hudson et al., 2014) showing that burst suppression is not likely to occur at this concentration, but that state transitions in the spectral characteristics of the LFP are frequently observed. Here we focused on the LFPs recorded using two laminar probes that sampled signals across all cortical layers. In half of the experiments, both electrodes were inserted into the right hemisphere: one in the primary visual area (V1) and the other in the motor cortex (M1) (*n* = 3) (Fig. 1*A*, left). In the other half of experiments, bilateral V1 recordings were performed (*n* = 4). Postmortem localization of electrodes (see Materials and Methods) in a representative experiment is shown in Figure 1*A* (right). Consistent with previous findings (Hudson et al., 2014), at 1% isoflurane, the power spectrum of the LFP fluctuated between several discrete states (Fig. 1*B*).

**Figure 2.**
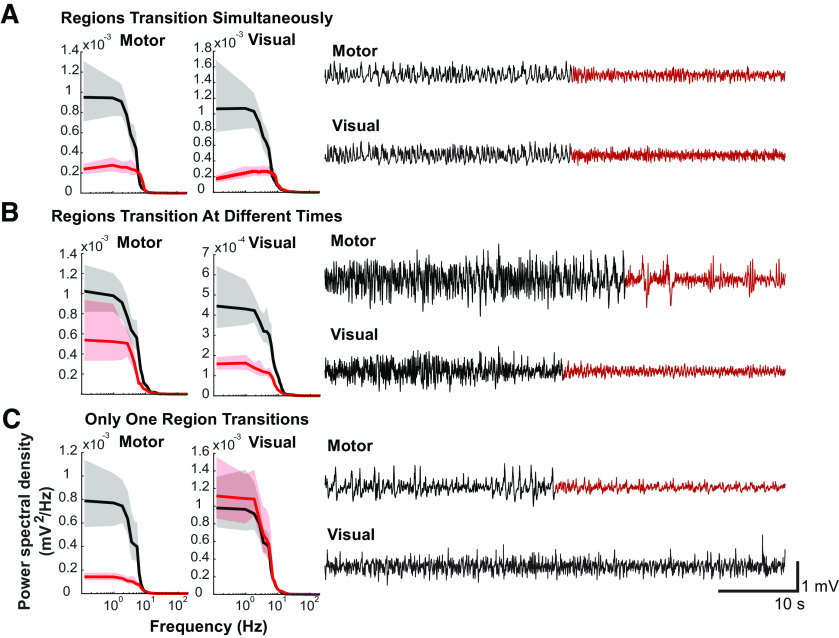
Examples of state transitions. ***A–C***, Right, LFP traces (1 min) recorded simultaneously from right M1 and V1. Visually apparent abrupt transitions in the character of the LFP are indicated by shifts of color from black to red. Left, Spectra computed from the red and black time periods, show that the abrupt switches in the features of the signals are associated with changes in the spectra. Shaded areas are 95% CIs computed from 1000 bootstraps. ***A***, An example where both M1 and V1 LFPs appear to change state simultaneously. ***B***, An example where both M1 and V1 signals change states but with an appreciable time delay (∼10 s). ***C***, An example where a state transition is observed in M1 but not in V1. In this case, for the purposes of computing the spectrum (left, red) in V1, the time segment highlighted in red for the M1 electrode was used.

State transitions can be readily identified by visual inspection of the raw LFP (Fig. 2). The top and bottom LFP traces show 1 min of recordings from a single M1 and V1 electrode, respectively. The accompanying spectra were calculated using a multitaper spectral estimate. These spectra were averaged across 2 s windows of LFP with a 1 s step size, sampled either from 8 to 2 s before transition (black, before transition) or from 2 to 8 s after the transition (red, after transition). Spectral estimates are shown as mean ± 95% CI computed from 1000 bootstraps. In some instances, state transitions occur approximately simultaneously in the motor and visual cortices (Fig. 2*A*). However, this was not always the case. For instance, Figure 2*B* shows an example of a state transition that occurs first in the visual cortex and, only after a delay of ∼10 s, is seen in the motor cortex. Thus, abrupt changes in the LFP characteristics need not occur simultaneously in different brain regions. Figure 2*C* shows a more extreme example of this phenomenon. A state transition is clearly seen in the motor cortex, but in the visual cortex, the LFP characteristics remain unchanged. These observations suggest that, while some state transitions may indeed be global, there is a previously unappreciated degree of independence between state fluctuations observed in the cortex during fixed anesthetic administration.

### Multitaper analysis and NMF extract states and their transitions across cortical layers and regions

**Figure 3.**
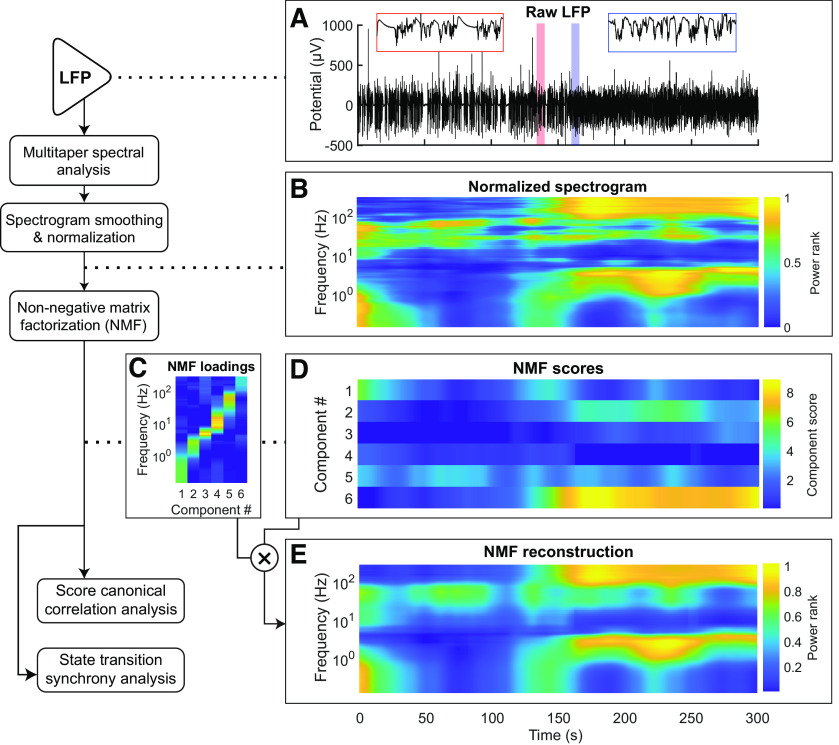
Schematic of LFP analysis, through NMF calculation. Left, Flowchart of analysis steps. ***A***, Five minutes of raw LFP signal centered around a state transition. Red and blue callout boxes zoom in on 6 s of LFP before and after a transition, respectively. ***B***, Spectrogram of LFP, computed using the multitaper method, after smoothing and rank-order normalization across time (see Materials and Methods). ***C***, ***D***, The loading (***C***) and score (***D***) matrices generated using NMF showing the spectral characteristics of each component and its relative contribution to the signal across time, respectively. The number of NMF components was optimized individually for each channel (see Materials and Methods). ***E***, Reconstruction of the normalized power spectrogram from NMF loadings and scores. As indicated, the reconstruction is simply the loading matrix multiplied by the score matrix.

**Figure 4.**
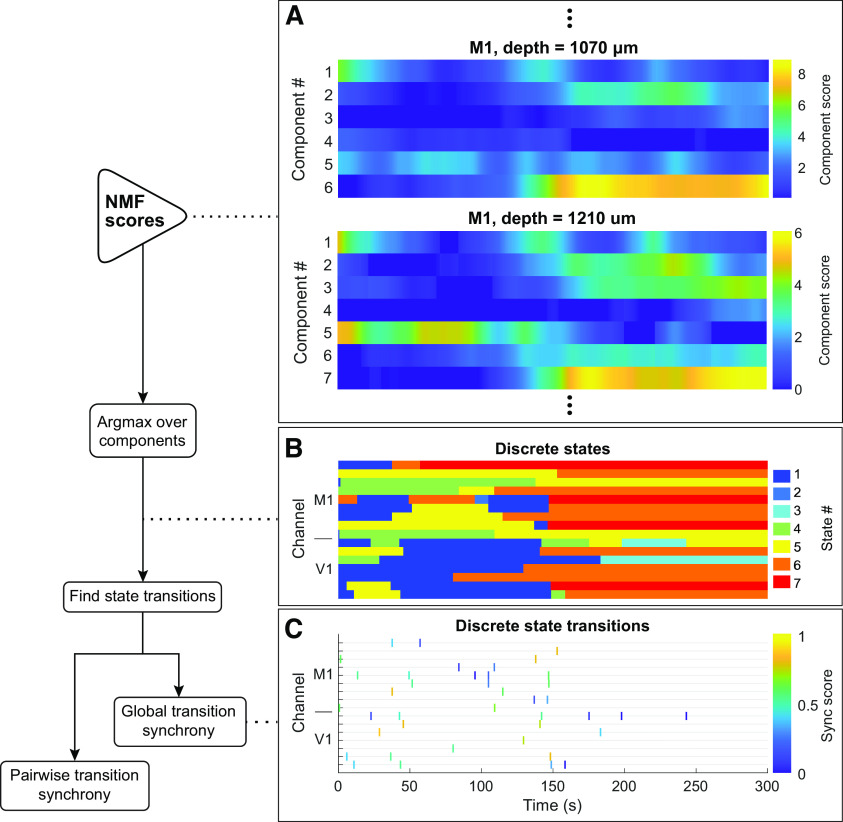
Schematic of NMF score analysis to define state transitions and synchrony. Left, Flowchart of analysis steps. ***A***, The NMF score matrix presented in Figure 3*D* (top) and another NMF score matrix from simultaneously collected LFP from a neighboring channel (bottom). While nearby channels share similar characteristics across time, they are not identical. Also, the two channels have different optimal “numbers” of components, since NMF was performed and optimized (see Materials and Methods) independently for each channel. ***B***, State assignments across example time window from 16 simultaneously recorded signals: 9 signals from an M1 electrode (top rows) and 7 from V1 (bottom rows). State # indicates the NMF component with the highest score in each time window, after removing state segments that were both short and ambiguous because of small score fluctuations (see Materials and Methods). ***C***, Raster plot of all transition times from the channels presented in ***B***. Transitions are colored according to their synchrony with transitions in all other channels (see Materials and Methods).

To quantify the degree of coupling between state transitions at different recording sites, we developed a methodology to automatically detect state transitions at the level of individual channels (see Materials and Methods), as illustrated in Figures 3 and 4. We then deployed this methodology to determine the degree to which transitions in different cortical sites are coupled. Briefly, wideband data were filtered between 0.1 and 300 Ηz to extract the LFP signal (Fig. 3*A*). LFP signals were converted to frequency domain using multitaper spectral analysis. Raw power spectra were then normalized such that the power contained in each frequency band was mapped onto a value between 0 (smallest observed power) and 1 (largest observed power) (Fig. 3*B*). Non-negative matrix factorization (NMF) was used to further decompose the signal into a set of loadings and associated scores across time (Fig. 3*C*,*D*). Figure 3*E* shows an example reconstruction of the spectrogram from NMF components; the error of the reconstruction was used to tune the number of NMF components for each channel (see Materials and Methods).

NMF can be thought of as a “soft” clustering algorithm. Previous work on state transitions under anesthesia (Hudson et al., 2014) and sleep (Gervasoni et al., 2004) used *k*-means clustering of the spectrograms to assign states to the brain. Our first approach to state assignment used a similar strategy: the index of the NMF component with the highest score in each time window was defined as the state of the LFP at each recording site (see Materials and Methods). This assumption was relaxed in subsequent stages of the analysis (see below). Figure 4*A* shows the score matrices for two different channels recorded simultaneously from two contacts along the same electrode in the motor cortex. The upper matrix is the same as Figure 3*D*, and the lower matrix was generated from data collected by a contact 140 μm deeper inside the cortex. These matrices resemble one another but are not identical. Figure 4*B* shows state classifications for 16 channels of simultaneously recorded data: nine from an electrode in M1 and seven from an electrode in V1. Some state transitions are observed around the same time in most of the electrodes. There are, however, many instances where a state transition is observed in just a subset of the recording sites.

**Figure 5.**
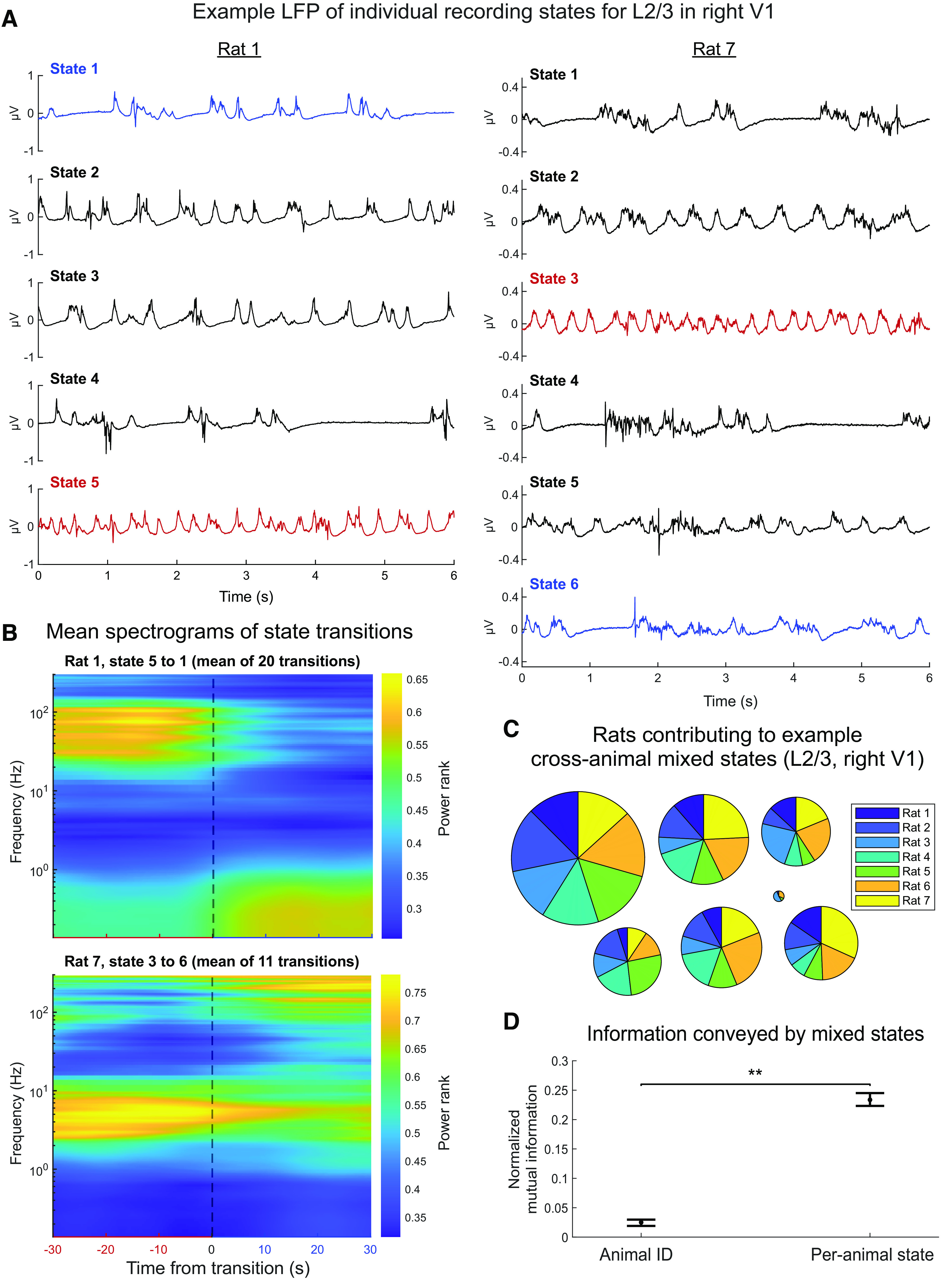
Automatic state classification captures distinct LFP patterns reproducible across animals. ***A***, Windows of LFP from an electrode in the center of layer 2/3 (370 μm) of 2 rats. To show the most prototypical examples, LFP windows were chosen such that the ratio of the highest NMF score (corresponding to the assigned state) to the sum of all scores is maximal. Red and blue traces represent the pre- and post-transition states of the most frequent state transition in each animal. ***B***, Mean normalized spectrograms around the most frequent state transition in recordings illustrated in ***A***. The number of transitions averaged to compute each spectrogram is shown in the title. ***C***, ***D***, In 500 independent replicates, spectra of random samples of 600 LFP windows from each state in the center layer 2/3 channel of each rat were concatenated, and “mixed states” were identified using the same NMF method as individual channels (see Materials and Methods). ***C***, Pie charts represent the fraction of LFP windows from each animal included in each mixed state in a randomly chosen replicate. The area of each pie chart is proportional to the total number of windows classified into the corresponding mixed state. All mixed states include windows from more than half of the animals. ***D***, Median and 95% CI for NMI across replicates of mixed state classifications with both animal IDs and per-animal state classifications (averaged across animals).

Before proceeding with the analysis of automatically detected states, we sought to determine whether they reflect differences in LFP characteristics akin to those in Figure 2. Figure 5*A* illustrates LFP patterns most strongly associated with each discrete state from 2 example rats (see Materials and Methods). In both rats, differences between states reflect the prevalence of characteristic patterns, such as slow oscillations, δ waves, and intermittent bursts of higher-frequency oscillations. These automatically detected states are essentially similar to those observed in other studies under similar experimental conditions (Hudson et al., 2014; Tort-Colet et al., 2021). To determine whether the automatically detected state transitions correspond to abrupt changes in the LFP spectra, we computed an average spectrogram around the most frequent state transition in each recording (Fig. 5*B*). As expected, we observe that the automatically detected state transition is associated with an abrupt change in the spectra.

Throughout this work, we defined states locally for each recording individually. Thus, by design, our analysis does not explicitly assume an essential similarity between different LFP states observed at different recording sites or in different animals. Nevertheless, traces shown in Figure 5*A* suggest that the state detection algorithm picks up similar patterns across different animals. To quantify the consistency of state assignments in different animals, we applied our state assignment algorithm to each recording individually (local states) and to data combined across animals (mixed states). If the local states were reproducible and consistent across animals, we expect mixed states to combine similar local states between multiple animals, without splitting local states apart. Figure 5*C* shows that, in most cases, each mixed state has approximately equal representation of the data from each recording. Figure 5*D* compares the NMI of mixed states with animal IDs to NMI between mixed and local states across 500 bootstraps. The median NMI with local states is significantly higher (*p* = 0.002), suggesting that each mixed state contains more information about the local state than the animal ID. Together, this analysis suggests that, while our methodology does not explicitly impose similarity in state assignments in different recordings, it picks out similar states across different animals and that these states correspond to known physiological characteristics of LFPs under isoflurane anesthesia.

One way to characterize the coupling between state transitions is to quantify the propensity of state transitions to occur simultaneously across different recording sites. Brain state transitions were defined as time points at which consecutive windows from the same channel have different brain state assignments (see Materials and Methods). Figure 4*C* shows an example of this analysis. There are many transitions that appear in only one or very few channels, while others appear to be more global. Figure 4*C* is a raster plot of transitions. The color of each line shows the synchrony score of that transition with all other channels (see Materials and Methods). Consistent with the observations in Figures 2 and 4*C*, the synchrony score reflects the fact that most state transitions are localized to a small subset of electrodes.

**Figure 6.**
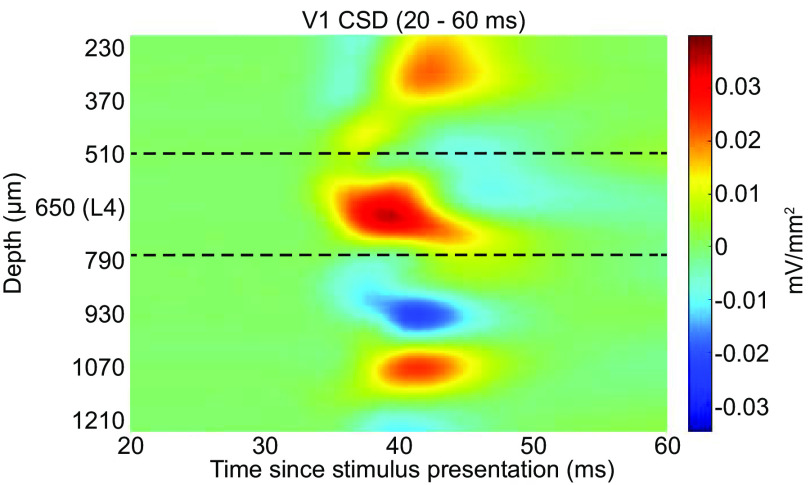
CSD computed for a representative V1 recording. Evoked potential was elicited using a brief green LED flash (see Materials and Methods). Dotted lines indicate the approximate boundaries of L4. Depth represents estimated depth from the cortical surface.

As we show below, coupling between state transitions depends on the cortical layer. Layer assignment in V1 was performed using CSD analysis computed immediately following brief light stimulus (see Materials and Methods). Figure 6 shows a representative example of CSD in V1 showing the stereotypical pattern of response to visual stimuli. The first current sink occurs ∼33 ms following stimulus presentation in L4. A short time after, additional sinks and sources appear above and below, revealing interlaminar communication. The channel where the initial sink occurred was defined as the center of L4. The dashed black lines in this figure indicate the approximate boundaries of L4 based on the average thickness of this layer in rats and the spacing between channels (Quairiaux et al., 2011; Einevoll et al., 2013; Self et al., 2013). In the motor cortex, we did not estimate the location of cortical layers directly. Instead, we estimated the depth of each recording electrode relative to the cortical surface.

### State transitions in different cortical sites exhibit weak synchrony

**Figure 7.**
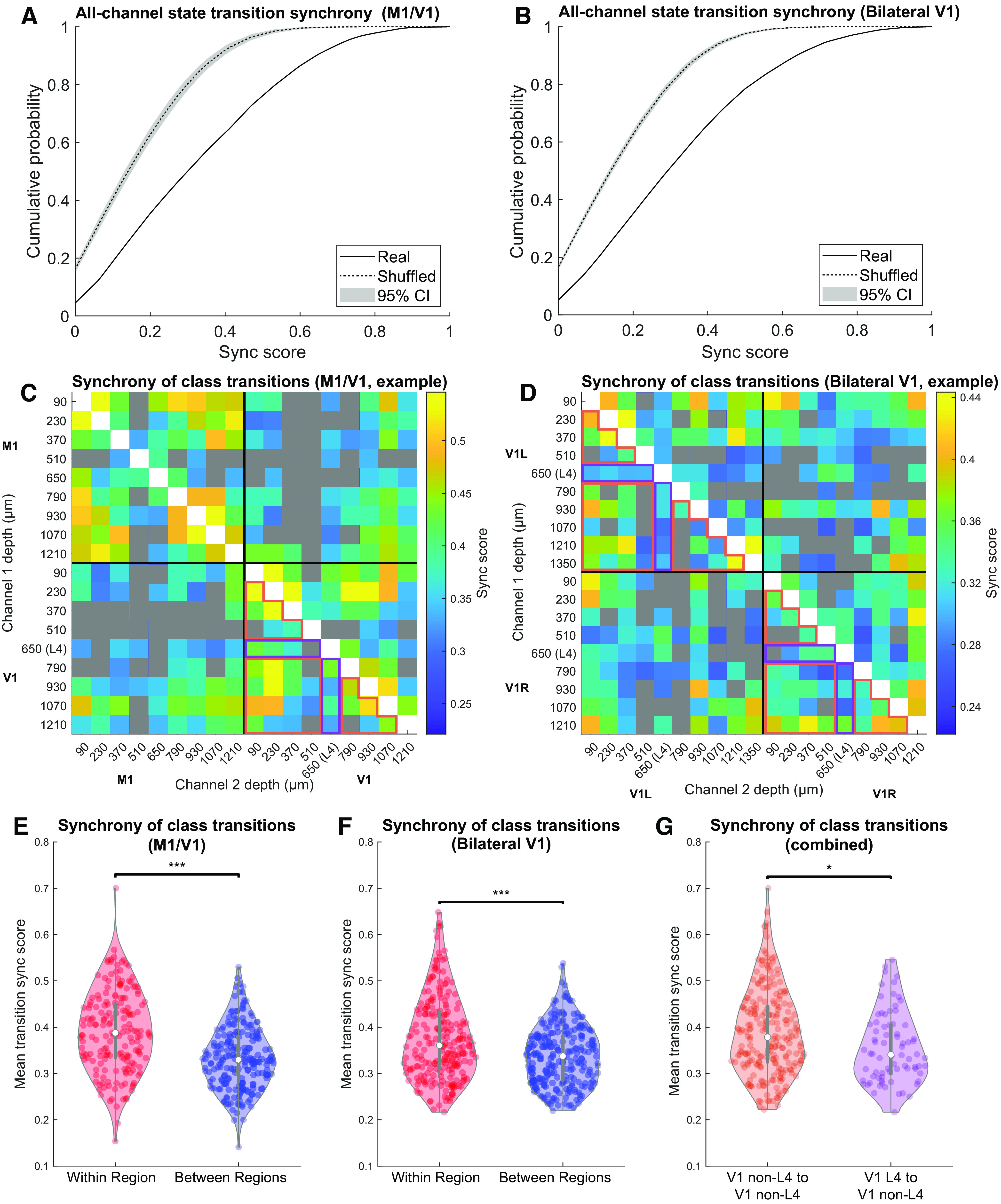
Transition synchrony between channels in the same anatomic region is higher than between channels in different regions. ***A***, ***B***, The cumulative distribution of SPIKE-synchronization (synchrony) scores across all channels, in real recordings (solid) and the median ± 95% CI of 1000 shuffled recordings (dotted), for M1/V1 experiments (***A***) and bilateral V1 experiments (***B***). ***C***, ***D***, Mean synchrony score across transitions for all channel pairs from a representative M1/V1 (***C***) and bilateral V1 (***D***) recording. Channel pairs whose synchrony scores were not significantly different from shuffled controls after Bonferroni correction are colored gray. ***E***, ***F***, Channel pairs in which both channels are in the same region (red) have higher synchrony scores than those in which the channels are in different regions (blue) for M1/V1 (***E***, *p* = 1e-7, permutation test) and bilateral V1 (***F***, *p* = 2e-7, permutation test) recordings. ***G***, Channel pairs in which one channel was within L4 and the other was not had lower synchrony scores than pairs in which neither channel was in L4 (*p* = 0.015, permutation test). Data included in these comparisons for the representative experiments are outlined in orange and purple, respectively, to highlight that only data from V1 electrodes were used. *p < 0.05; ***p < 0.001.

We used three different analytical techniques to quantify the tendency of oscillatory states and the transitions between them to be coordinated across recording sites. Each technique relies on a different set of assumptions and was performed on a different feature of the data. First, we quantified the synchrony of transitions, as demonstrated in Figure 4 (see Materials and Methods). Figure 7*A*, *B* shows the cumulative distribution of synchrony scores (solid curves) computed over all channel pairings and across all animals (M1/V1: 3 animals, 16-18 electrodes/animal, median of 99 transitions/electrodes/animal; bilateral V1: 4 animals, 15-19 electrodes/animal, median of 175.5 transitions/electrode/animal).

In order to compare the synchrony scores (Fig. 7*A*,*B*) to those expected by chance, we generated shuffled datasets constrained to have the same state transition statistics. This was accomplished by simulating a Markov process defined by the state transition probability matrix derived from state assignments for each recording (see Materials and Methods). This control preserves the statistics of each recording site, while destroying any coordination between them. The cumulative distributions of the synchrony scores obtained in these shuffled controls are shown in Figure 7*A*, *B* (dotted curves; shading shows 95% CIs computed over 1000 shuffled datasets). Both in the experiments involving M1 and V1 (Fig. 7*A*) and in those involving bilateral V1s, we find that the synchrony score is consistently higher than expected by chance (*p* < 0.001, *z* test based on means of shuffled datasets). Despite this large deviation from the null hypothesis, state transitions do not typically occur at the same time in different cortical sites (mean synchrony score ≈ 0.35 for both M1/V1 and bilateral V1 recordings). This implies that while state transitions observed across different cortical sites are not completely independent, coupling between channels is weak.

Data in Figure 7*A*, *B* aggregate the transition synchrony scores calculated between all channel pairs: both pairs of channels in the same cortical region and those located in different cortical regions. We hypothesized that, because most cortical connectivity is local, nearby electrodes would tend to have a higher propensity to change state at the same time. Figure 7*C–F* shows that state transitions are indeed more synchronous between electrodes within a cortical region than between regions. Figure 7*C*, *D* shows synchrony scores between all channel pairs in a representative pair of experiments: an M1/V1 experiment (Fig. 7*C*) and a bilateral V1 experiment (Fig. 7*D*). Pairs with scores that did not reach significance compared with the shuffled datasets, after Bonferroni correction for multiple comparisons, are shown in gray. Across all experiments, 57.0% of channel pairs from M1/V1 experiments and 80.2% of pairs from bilateral V1 experiments had significantly synchronous transitions at the corrected *p* < 0.05 level. The synchronization scores for all channel pairs from all experiments are quantified in Figure 7*E*, *F*, for M1/V1 and bilateral V1 experiments, respectively. Both panels show the synchrony scores for within-region channel pairs (red) and between-region channel pairs (blue). In both types of recordings, within-region pairs had significantly larger synchrony scores than between-region pairs (*p* = 1e-7 for M1/V1 and *p* = 2e-7 for bilateral V1, compared with 10^7^ random permutations of the relevant channels) (see Materials and Methods).

L4 is the thalamic input layer and has fewer horizontal connections than the supragranular or infragranular layers (Zilles and Palomero-Gallagher, 2017). To test whether layer organization affects transition synchrony, from each V1 recording (in which L4 was identified using CSD), we separated channel pairs in which one channel was in L4 from pairs in which neither channel was in L4. Figure 7*G* presents synchrony scores from all channel pairs from all experiments in which one channel was in L4 and the other was not (purple) and all channel pairs from all experiments in which neither channel was in L4 (orange). In Figure 7*E*, *F*, the specific channel pairs that were included in the “L4” and “non-L4” groups are outlined in purple and orange, respectively. We found that synchrony between channel pairs with one channel in L4 tended to be lower than between pairs in which neither channel was in L4 (*p* = 0.015, compared with 10^7^ random permutations of the relevant channels) (see Materials and Methods). Therefore, transition times in channels from L4 tend to be relatively uncoupled from the specific timing of transitions in channels from other layers. This observation suggests that it is unlikely that core thalamocortical input is the principal driver of state transitions in the cortex. If it were, one would expect that the thalamic input layer (L4) would transition in synchrony with the rest of the cortex. Therefore, these results imply different mechanisms, such as corticocortical interactions and/or matrix projections from the thalamus, are likely responsible for the timing of these spatially localized transitions.

Our final analysis using synchrony scores was performed to build on these L4 results and determine whether the type of subcortical input to a cortical region has an influence on transition synchrony. It is typically assumed that switches of the oscillatory activity in the cortical LFP critically involve interactions with the thalamus (Steriade et al., 1993a, 1994; Contreras and Steriade, 1997; Schiff, 2008; Liu et al., 2015; Herrera et al., 2016). In light of this, one may expect two regions receiving similar thalamic input to exhibit greater synchrony of state transitions than two regions that interact with the thalamus in different ways. Therefore, we tested whether between-region comparisons for the bilateral V1 experiments had higher synchrony scores than the between-region comparisons for the M1/V1 experiments. Contrary to our hypothesis, we were not able to detect any increase in synchrony scores calculated between the bilateral V1s relative to M1/V1 experiments (*p* = 0.35, percentile bootstrap over channels) (see Materials and Methods).

### Discrete states in different cortical sites have weak correspondence

We now shift our focus away from the timing of state transitions and quantify the consistency of LFP-defined states at different sites. We accomplish this using NMI, a measure of the amount of information obtained about one random variable by observing another random variable (see Materials and Methods). In our case, these random variables are the time series of discrete states of two channels. High NMI between these time series represents a large reduction in uncertainty about the state in channel *j* given the state in channel *i*. Two channels do not need to be in the same brain state to have high mutual information; indeed, since states are defined for each channel independently, there is no definition of different channels being in the “same” state.

**Figure 8.**
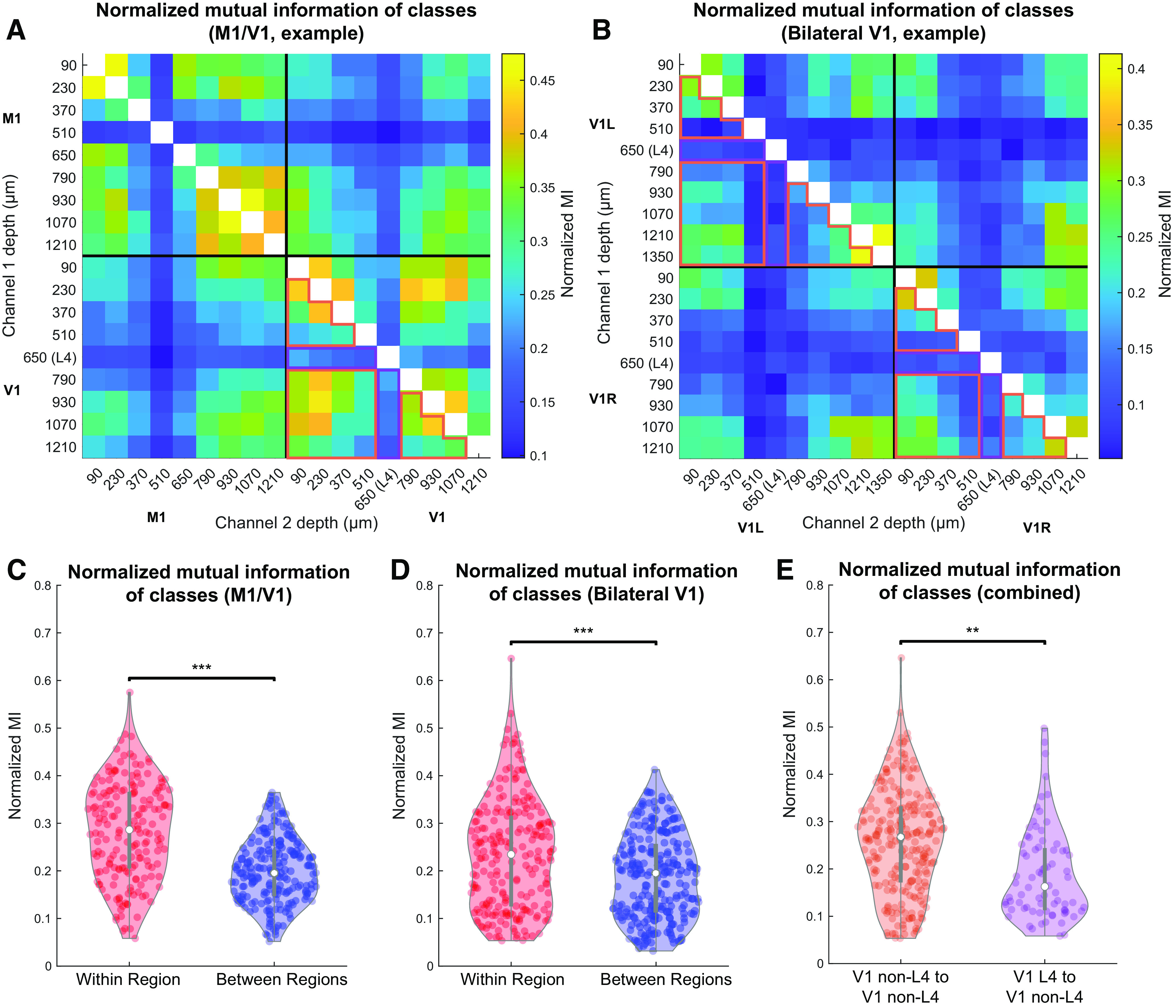
NMI between channels in the same anatomic region is higher than between channels in different regions. ***A***, ***B***, NMI between state assignment vectors for all channel pairs from a representative M1/V1 (***A***) and bilateral V1 (***B***) recording. All NMI values are significantly different from shuffled controls after Bonferroni correction. ***C***, ***D***, Channel pairs in which both channels are in the same region (red) have higher NMI than those in which the channels are in different regions (blue) for M1/V1 (***C***, *p* = 1e-7, permutation test) and bilateral V1 (***D***, *p* = 1e-7, permutation test) recordings. ***E***, Channel pairs in which one channel was within L4 and the other was not had lower NMI than pairs in which neither channel was in L4 (*p* = 0.002, permutation test). Data included in these comparisons for the representative experiments are outlined in orange and purple, respectively, to highlight that only data from V1 electrodes were used. **p < 0.01; ***p < 0.001.

Figure 8*A*, *B* shows the NMI between all channel pairs in the same representative M1/V1 and bilateral V1 experiments as those in Figure 7*C*, *D*; 81.9% of channel pairs from M1/V1 experiments and 96.9% of pairs from bilateral V1 experiments had NMI that was significantly higher than for shuffled data, after Bonferroni correction for multiple comparisons (*z* test based on distribution of shuffled data). The summary of NMI across all animals is shown in Figure 8*C*, *D*, for M1/V1 and bilateral V1 experiments, respectively. In both types of recordings, within-region channel pairs had significantly higher NMI than between-region pairs (*p* = 1e-7 for M1/V1 and *p* = 1e-7 for bilateral V1, compared with 10^7^ random permutations of the relevant channels) (see Materials and Methods). While for most channel pairs NMI was higher than for a shuffled dataset, the amount of information about the state of one channel contained in the state of another was small. NMI varies between 0 and 1, where 1 denotes that the two channels carry identical information. Yet, even in a pair of channels within a single cortical region, the mean NMI is ∼0.3. One way to interpret this statistic (see Materials and Methods) is that no more than 15% of the combined information carried by the states of any two channels is redundant. Thus, most of the information about the state of one channel cannot be extracted from observing the state of a nearby channel in the cortex.

As with transition synchrony, we did not detect a higher mean NMI in left/right V1 channel pairs compared with M1/V1 channel pairs (*p* = 0.70, percentile bootstrap over channels) (see Materials and Methods). Additionally, as with the transition synchrony analysis, pairs including a channel in L4 did have lower NMI than pairs where neither channel was in L4 (*p* = 0.002, compared with 10^7^ random permutations of the relevant channels) (see Materials and Methods). These results show not only that channels from the same brain region are more likely to undergo transitions at the same time, but also that the broader structure of these state assignments across the entire recording is more similar in channels from the same region. Furthermore, the conclusions regarding the differences between L4 and other cortical layers are consistent between synchrony and mutual information analyses.

### Full compressed spectrograms of different sites have moderate correspondence, depending on distance and cortical layer

In the previous analyses, to generate a single value description of activity across time, we defined brain state as the NMF loading with the highest score in each time window. This method was convenient for comparing synchrony of transitions and mutual information of state sequences. Parcellation of the LFP signals into discrete states is also supported by previous work (Hudson et al., 2014). However, reducing the LFP to a single value eliminates much of the information in the original signal. In order to incorporate more of this information, rather than collapsing the LFP signal to a single value, we used the vector of NMF scores for the LFP in each temporal window directly. Each score vector, once multiplied through by the appropriate loading matrix (see Materials and Methods; Fig. 3), yields a good approximation of the actual spectrum of the LFP in that time window.

**Figure 9.**
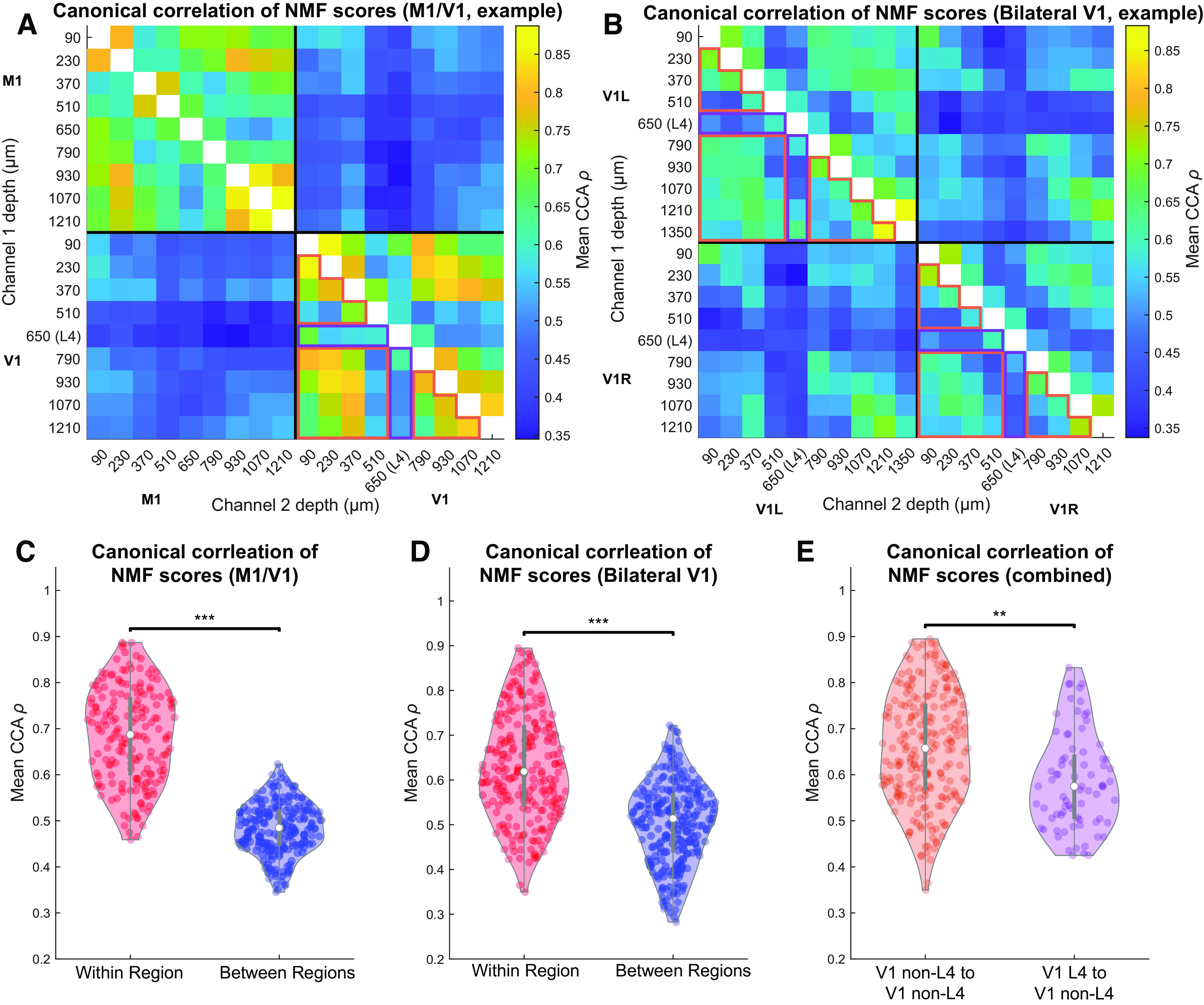
CCA reveals higher correspondence of overall activity between channels in the same anatomic region than between channels in different regions. ***A***, ***B***, CCA measure on NMF scores for all channel pairs from representative M1/V1 (***A***) and bilateral V1 (***B***) recordings. ***C***, ***D***, Channel pairs in which both channels in the same region (red) have higher NMF score correspondence than those in which the channels are in different regions (blue) for M1/V1 (***C***, *p* = 1e-7, permutation test) and bilateral V1 (***D***, *p* = 1e-7, permutation test) recordings. ***E***, Channel pairs in which one channel was within L4 and the other was not had lower NMF score correspondence than pairs in which neither channel was in L4 (*p* = 0.001, permutation test). Data included in these comparisons for the representative experiments are outlined in orange and purple, respectively, to highlight that only data from V1 electrodes were used. **p < 0.01; ***p < 0.001.

To test for correlated fluctuations in the spectral features of LFPs at different cortical sites, we applied CCA to the pair of score matrices derived from each pair of channels. High canonical correlation indicates a close linear relationship between two sets of variables. The mean of the vector ρ of canonical correlations between all pairs of canonical variables was calculated to give a measure of overall state similarity that is invariant to invertible linear transformations of each channel's state space (Alpert and Peterson, 1972). Figure 9*A*, *B* shows the CCA similarity measure for all channel pairs from the same representative M1/V1 and bilateral V1 experiments that have been shown previously. All channel pairs from both M1/V1 and bilateral V1 experiments had significantly higher CCA similarities than for shuffled data, after Bonferroni correction for multiple comparisons (*z* test based on distribution of shuffled data). The summary of CCA similarity across all animals is shown in Figure 9*C*, *D*. These results are very similar to those for transition synchrony and NMI and show that, in both types of recordings, within-region channel pairs had significantly higher CCA similarities than between-region pairs (*p* = 1e-7 for M1/V1 and *p* = 1e-7 for bilateral V1, compared with 10^7^ random permutations of the relevant channels) (see Materials and Methods). Furthermore, as with the previous measures, channel pairs including a channel in L4 had lower CCA similarities than pairs in which neither channel was in L4 (*p* = 0.001, compared with 10^7^ random permutations of the relevant channels) (see Materials and Methods). We did not detect a higher mean CCA similarity in left/right V1 channel pairs compared with M1/V1 channel pairs (*p* = 0.12, percentile bootstrap over channels) (see Materials and Methods).

### Global brain state is low-dimensional, despite weak pairwise interactions

All results shown up until this point were calculated on pairs of channels for which state assignments were computed independently. What we have shown is that channels within the same cortical region tend to be more similar in their activity patterns and state transition times than channels from different cortical regions. However, close inspection of the results shows that, even for the channel pairs within the same cortical region, only about one-third of the information contained within the discrete state sequences is shared between channels (Fig. 8*C*). For channel pairs from different cortical regions, the amount of mutual information in state sequences is even lower. This weak coupling between channels could imply that spatially restricted regions of the brain act independently of one another and there is no discernable global state of the brain at any given time. Alternatively, it is possible that this weak coupling between channels, *en masse*, gives rise to a complex, global state of activity that is differently expressed in the oscillation patterns of spatially restricted regions of cortex. In this final analysis, we sought to directly distinguish these possibilities by characterizing the global brain state. In this case, global brain state is defined as the spectra of all recorded signals concatenated together, in a similar fashion to that used by Hudson et al. (2014). In a key distinction from the previous work, we defined global macroscopic dynamics from the simplified dynamics observed at each recording site. This was accomplished by first concatenating the NMF score vectors from all simultaneously recorded channels at each time point into a single vector that encodes the joint state of all channels. The resulting full matrix of joint states over time was then subjected to principal component analysis.

**Figure 10.**
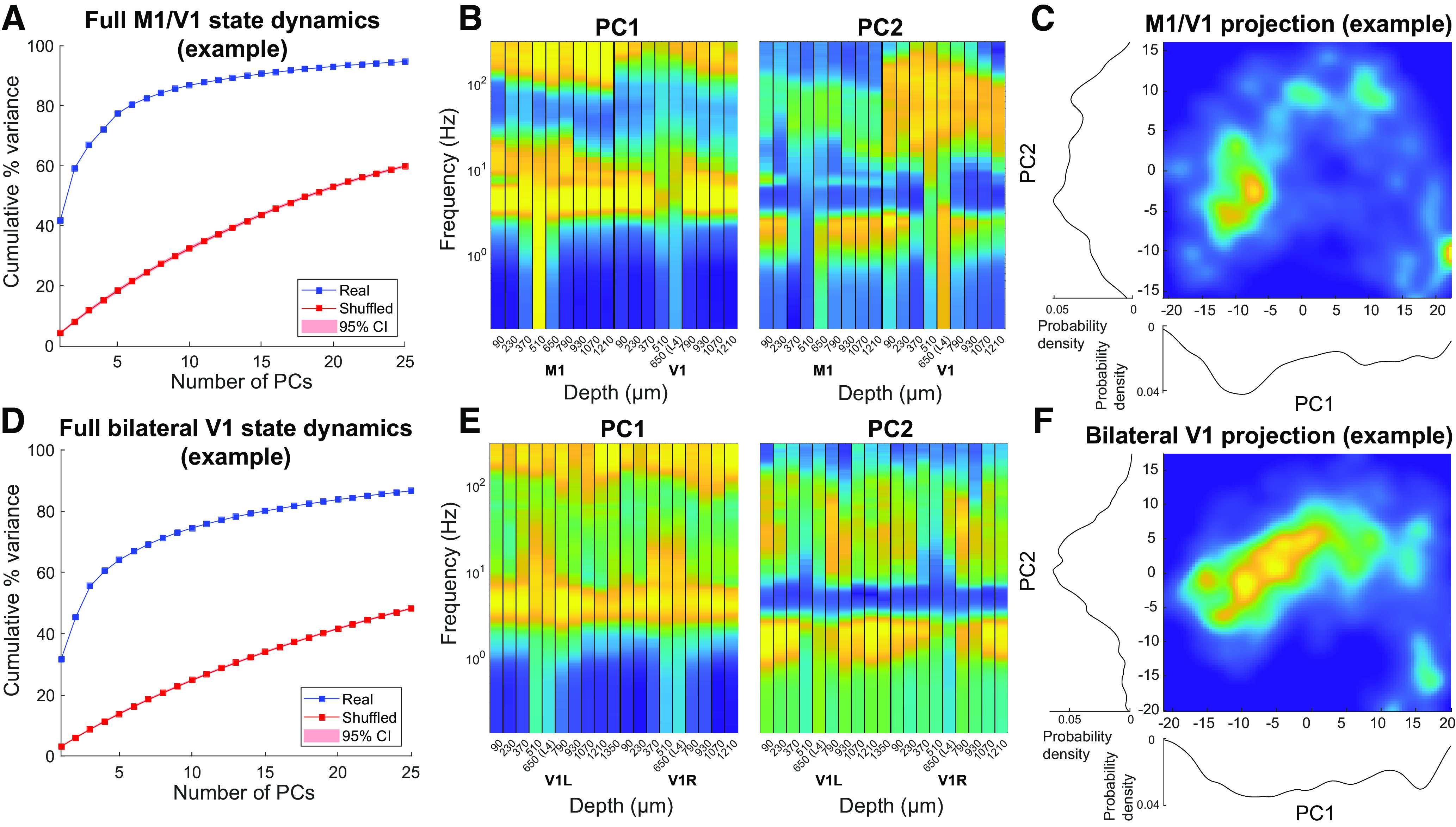
Weakly correlated fluctuations in different cortical sites give rise to highly correlated cortical states. NMF scores from all recorded channels were concatenated into a single-state vector (median dimension across recordings = 106) and subjected to principal component analysis. Fraction of total variance as a function of number of PCs is shown for M1/V1 (***A***) and bilateral V1 (***D***) example recordings (blue). Shuffled surrogates (see Materials and Methods) were subjected to the same analysis (red). ***B***, ***E***, Loadings of the top two principal components, mapped back from each channel's NMF components to frequencies, for the two representative recordings. This projection reveals consistent differences between M1 and V1 (***B***) but is relatively consistent across bilateral V1s (***E***). In both instances, layer 4 is distinct from supragranular and infragranular layers. ***C***, ***F***, Histograms of the data projected onto the top two PCs for the representative M1/V1 (***C***) and bilateral V1 (***F***) recordings. In both instances, the distribution of data are multimodal, suggesting the presence of discrete global cortical states.

We found that all but one recording required 10 or fewer components to account for 80% of the variance in the concatenated NMF score matrices, which ranged in dimensionality from 91 to 136. The recording that required >10 components required 15 components to reach the same threshold. This is far outside the 95% CI of expected cumulative explained variance, computed on Markov-shuffled controls, which ignore weak pairwise correlations between fluctuations in different channels (Fig. 10*A*,*D*). These results demonstrate that widespread weak coupling is sufficient to give rise to a highly correlated global state. Figure 10*B*, *E* shows the loadings onto channels and frequencies (mapped back from corresponding NMF loadings) for the top two principal components of a representative M1/V1 and bilateral V1 recording, respectively. These data offer qualitative evidence that the global state is differentially reflected in different regions and layers of the cortex. For example, the loadings of the second principal component (PC2) of the M1/V1 recording in Figure 10*B* show that, while there is high power in the higher frequencies for the V1 channels, the same is not true in the M1 channels. In contrast, Figure 10*E* shows that the loadings of PC1 of the bilateral V1 recording onto all channels of both electrodes are fairly uniform, except for in channels near L4 where there is higher power in the lowest frequency bands. Figure 10*C*, *F* shows histograms of all samples from these representative recordings projected onto the first two principal components. Although more than two dimensions would be necessary to fully visualize the landscape of the global dynamics, even in this limited projection, a clustered pattern is visible, similar to previous results (Hudson et al., 2014). These data suggest that global brain states comprise regionally distinct oscillation patterns that are weakly coupled with one another. Remarkably, these results show that discrete transitions between global cortical states (Hudson et al., 2014; Ballesteros et al., 2020; Patel et al., 2020) under a fixed anesthetic concentration arise from the multitude of weakly coupled local fluctuations.

## Discussion

Here we analyzed local synchrony of neural oscillations between recording sites at a fixed anesthetic concentration to elucidate how global states are coordinated. According to three complementary measures, oscillatory patterns are only weakly coupled between most pairs of cortical sites. However, combining data across all channels reveals that macroscopic activity patterns are nevertheless low-dimensional. We also demonstrate that the strength of the interactions between recording sites depends on the cortical regions and layers being compared. Specifically, sites in different regions tend to be less synchronized than sites in the same region. Additionally, L4, the primary thalamic input layer, is less synchronized with other cortical layers than those layers are with each other. Together, these results argue that abrupt state transitions are strongly influenced by local interactions within the thalamocortical network.

Several studies using mechanistically distinct anesthetics and different model organisms converge on the fact that on the neurophysiological level, general anesthesia consists of a set of discrete global activity states (Chander et al., 2014; Hudson et al., 2014; Ballesteros et al., 2020; Lee et al., 2020; Patel et al., 2020). Intuitively, this may seem to imply that different cortical sites must switch states synchronously. In contrast to this intuition, we show that local state fluctuations are only weakly correlated, but the multitude of such weak correlations is sufficient to constrain brain activity to a few discrete global states (Fig. 10). There is no contradiction between quasi-independent local fluctuations and highly correlated global phenomena. For example, weak pairwise correlations in spike timing have been shown to produce highly correlated neuronal ensembles (Schneidman et al., 2006) in diverse systems (Tang et al., 2008; Yu et al., 2008; Ohiorhenuan et al., 2010; Tkačik et al., 2014). Unlike these authors, we did not explicitly fit a maximum entropy model to our data because we only sampled LFPs from two brain regions per animal. To test whether a maximum-entropy model of pairwise correlations is sufficient to explain global cortical synchrony (as in Tang et al., 2008; Ohiorhenuan et al., 2010), future work should sample LFP fluctuations more densely across the cortex.

Our findings have immediate implications for monitoring the state of anesthesia. In most clinical settings, anesthetic state is defined on the basis of a few frontal EEG electrodes (Mashour, 2006). If state transitions are only weakly correlated, it is not possible to reliably infer the global state by observing a single location. This may be one reason for the failure of anesthetic monitors to detect intraoperative awareness with recall (Avidan et al., 2011). While awareness with recall is the most dreaded anesthetic complication, a variety of states of partial consciousness have been observed under anesthesia. Some patients can intermittently respond to verbal commands (Tunstall, 1977; Sanders et al., 2017) but do not exhibit any recall. Other patients recall vivid experiences under anesthesia wholly unrelated to the events in the operating room, despite unresponsiveness (Sanders et al., 2012). The fragmented nature of some such states may plausibly arise from weak coupling between different brain regions. Some cortical sites may transiently enter wake-like activity states, giving rise to fragments of complete conscious experience. In contrast, full awareness with recall may require a global state transition.

Since we focused on activity under anesthesia, fluctuations in the power of slow oscillations (<1 Hz) were particularly relevant to state transitions. Our results support the hypothesis that, outside of L4, these fluctuations are coordinated primarily through corticocortical interactions. Slow oscillations have been shown previously to be primarily generated by corticocortical synaptic mechanisms (Steriade et al., 1993b; Sanchez-Vives and McCormick, 2000). Corticocortical interactions are also thought to underlie the synchronization of slow waves across the cortex (Amzica and Steriade, 1995). Here, using three distinct analysis methods, we consistently find that fluctuations in L4 are especially decoupled from those in the infragranular and supragranular layers. L4 neurons are most directly affected by spatially localized core projections from the thalamus, whereas infragranular and supragranular neurons are primarily driven by corticocortical connections and matrix projections from the thalamus (E. G. Jones, 2001). While anesthetics suppress both core and matrix thalamocortical inputs, they primarily suppress corticocortical connectivity (Raz et al., 2014). Thus, the local nature of state transitions involving slow oscillations in the anesthetized brain is most likely because of weakened corticocortical connections, although an effect on matrix thalamocortical projections cannot be completely ruled out. The distinctiveness of L4 fluctuations may then be parsimoniously explained by anesthetics having a relatively small effect on core thalamocortical projections (Raz et al., 2014).

Our results are less consistent with a model where cortical state transitions are uniformly driven by deep nuclei. Transitions between slower (<4 Hz) and faster EEG oscillations are thought to arise from interactions of the thalamocortical networks with modulation from cholinergic (Steriade, 2004), noradrenergic (Vazey and Aston-Jones, 2014), and other nuclei in the brainstem and basal forebrain (B. E. Jones, 2003). Activity within these various arousal-promoting nuclei is, in turn, coordinated by a group of medullary neurons, activation of which can trigger prompt awakening from deep states of anesthesia (Gao et al., 2019). Under anesthesia, changes in the firing rate of these medullary neurons coincide with changes in the spectral characteristics of the cortical LFP (Gao et al., 2019). Thus, the spontaneous fluctuations of LFP between slower and faster oscillations may be mediated in part by fluctuations in activity within modulatory nuclei. However, most arousal nuclei project broadly to the thalamus and cortex (B. E. Jones, 2003). If fluctuations in the state of the LFP were entirely driven by these projections, the fluctuations should be coherent across the cortex. Furthermore, we would expect the state fluctuations within a cortical region to be very strongly coupled. Instead, we observe only weak coupling. Thus, while modulatory nuclei may bias the cortex toward a particular state, the activity at each cortical site is likely also strongly influenced by interactions within the thalamocortical network.

Both sleep and anesthesia consist of several discrete states characterized by distinct patterns of oscillations in the cortex and thalamus (Saper et al., 2010). Furthermore, neurophysiological mechanisms underlying these states and transitions between them are common to both settings (Steriade et al., 1993b; Steriade and Amzica, 1998). For instance, many diverse anesthetics promote activity in sleep-active subcortical nuclei and suppress activity in wake-active nuclei (Nelson et al., 2002; Moore et al., 2012; Zhang et al., 2015; Jiang-Xie et al., 2019). Based on single-neuron and EEG recordings, it has long been hypothesized that sleep stages are global and that switching mechanisms prevent multiple stages from occurring simultaneously in different brain regions (Lu et al., 2006; Saper et al., 2010). However, analyses at the mesoscopic levels of neuronal populations and local fields show evidence of local sleep stage transitions (Poulet and Petersen, 2008; Nir et al., 2011; Vyazovskiy et al., 2011). For example, a task that elicits activity in specific cortical regions subsequently increases slow-wave activity only in these cortical regions (Huber et al., 2004), demonstrating that slow-wave oscillations can be locally generated. The degree of synchrony of sleep/wake stage transitions between cortical locations has not been systematically quantified. Because sleep is strongly influenced by both homeostatic and circadian drives, it may be challenging to disentangle these global influences from the local interactions between different sites in the cortex. In contrast, anesthesia allows us to study brain state switches in the absence of these drives. Based on our results, we hypothesize that globally coordinated shifts in cortical activity arise from weakly interacting local state switches during both sleep and anesthesia, but future work should investigate whether the underlying mechanisms are truly similar in both settings.

While our results show clear evidence that fluctuations in the LFP characteristics are only weakly coupled, the specific network mechanisms that control the coupling strength cannot be directly inferred from these data. For instance, while it is known that modulatory nuclei project broadly throughout the cortex, some regional differences in the projection patterns are well known to exist (Loughlin et al., 1982; B. E. Jones and Yang, 1985; Jones, 2003). Similarly, there is a new appreciation for the fact that multiple thalamic nuclei converge on the same cortical areas and project to all cortical layers (Muñoz-Castañeda et al., 2021). The role of subcortical systems in coordinating state fluctuations in different cortical sites should be examined in future work by performing simultaneous recordings in the cortex, thalamus, and the reticular activating system. Furthermore, while we here compared recordings made in V1 and M1, to better characterize the dependence of coupling strength on cortical distance, 3D recordings across cortical layers and in nearby sites should be performed in future work.
